# Uptake and Transformation of Methylated and Inorganic Antimony in Plants

**DOI:** 10.3389/fpls.2018.00140

**Published:** 2018-02-13

**Authors:** Ying Ji, Adrien Mestrot, Rainer Schulin, Susan Tandy

**Affiliations:** ^1^Department of Environmental System Science, Institute of Terrestrial Ecosystems, ETH Zürich, Zürich, Switzerland; ^2^Institute of Geography, Faculty of Science, University of Bern, Bern, Switzerland

**Keywords:** trimethyl antimony(V), antimonate, antimonite, plant, shooting ranges, Sb(V), Sb(III), TMSb

## Abstract

Used as a hardening agent in lead bullets, antimony (Sb) has become a major contaminant in shooting range soils of some countries including Switzerland. Soil contamination by Sb is also an environmental problem in countries with Sb-mining activities such as China and Bolivia. Because of its toxicity and relatively high mobility, there is concern over the risk of Sb transfer from contaminated soils into plants, and thus into the food chain. In particular there is very little information on the environmental behavior of methylated antimony, which can be produced by microbial biomethylation of inorganic Sb in contaminated soils. Using a new extraction and high-performance liquid chromatography inductively coupled plasma mass spectrometry (HPLC-ICP-MS) method, we investigated antimony speciation in roots and shoots of wheat, fescue, rye, and ryegrass plants exposed to trimethyl antimony(V) (TMSb), antimonite (Sb(III)), and antimonate (Sb(V)) in hydroponics. The total root Sb concentrations followed the order Sb(III) treatment > Sb(V) treatment > TMSb treatment, except for fescue. Shoot Sb concentrations, however, did not differ among the three treatments. In the Sb(V) treatment small quantities of TMSb were found in the roots, whereas no TMSb was detected in the roots of Sb(III)-treated plants. In contrast, similar concentrations of TMSb were found in the shoots in both inorganic Sb treatments. The results indicate that biomethylation of Sb may occur in plants. In the TMSb treatment TMSb was the major Sb species, but the two inorganic Sb species were also found both in shoots and roots along with some unknown Sb species, suggesting that also TMSb demethylation may occur within plant tissues. The results furthermore indicate that methylated Sb is more mobile in plants than inorganic Sb species. Knowledge about this is important in risk assessments of Sb-contaminated sites, as methylation may render Sb more toxic than inorganic Sb, as it is known for arsenic (As).

## Introduction

Antimony (Sb) is a metalloid in Group V of the periodic table. Although it has also been defined as a priority pollutant in the 1970s (EU, 1976; U.S. EPA, 1980), it has received much less attention than its sister element arsenic (As) in the same group. Exposure to antimony can cause health problems such as joint and muscle pain, diarrhea, and vomiting (ATSDR, 2002). Single doses of 300 mg antimony kg^−1^ ingested as potassium antimony tartrate were found to kill rats (ATSDR, 2002). Antimony has an increasing range of applications, and its emissions into the environment, which mainly result from anthropogenic activities such as mining, traffic, industrial applications and shooting, continue to increase (He et al., 2012; Wan et al., 2013). Used as a hardening agent in lead bullets, Sb released from corroding bullets into shooting range soils is an environmental problem of particular concern in some countries. In Switzerland, between 10 and 25 t Sb is deposited yearly in shooting range soils, while 21 t was deposited in small arms firing ranges of Norway in the year 2000, and an annual Sb load of 1,900 t was estimated to enter soil in the United States through shooting (Wan et al., 2013). In other countries such as China and Bolivia, mining activities are a major source of soil contamination by Sb (Fontúrbel et al., 2011; He et al., 2012).

There are two stable oxidation states of Sb in the environment, Sb(III) and Sb(V). Under aerobic conditions, the dominant species is Sb(V) occurring as the anion ${\text{Sb}(\text{OH})}_{6}^{-}$ in soil solution, while under reducing conditions it is Sb(III) occurring as Sb(OH)_3_. Many soils are periodically or occasionally flooded or waterlogged (Boyer, 1982; Merot et al., 1995; Dear et al., 2003), so that redox conditions can vary between aerobic to anaerobic due to a lack of oxygen. A change in redox state from Sb(V) to Sb(III) can have a strong effect on Sb uptake by plants (Wan et al., 2013), which may be due to uptake of different Sb species along different pathways. In plants growing on shooting ranges and mining soils, Sb concentrations were found to range from 1.15 to over 1,000 mg kg^−1^ DW (Baroni et al., 2000; Casado et al., 2007; Dominguez et al., 2008). In their review of studies on Sb accumulation in plants, Tschan et al. (2009b) found a linear relationship between plant Sb and soluble soil Sb concentrations, which held over five orders magnitude, although with a lot of scatter. There is little information about the uptake mechanisms of inorganic antimony species by plants. Sb(III) is assumed to enter plant roots through the same pathway as arsenite (As(III)), as both hydroxide molecules share a similar structure, which is via transporters belonging to the family of aquaporins (Li et al., 2016). One of these transporters is the nodulin 26-like intrinsic protein NIP 1;1, which was found in *Arabidopsis thaliana* (Kamiya and Fujiwara, 2009). Even less is known about the uptake pathway of Sb(V). Due to the different molecular structure of arsenate hydroxide and antimonate hydroxide, their uptake pathways are probably not the same. It was suggested that it is taken up by roots primarily via the apoplastic pathway and may enter cells via anion transporters (Tschan et al., 2009a).

Inorganic Sb can be biomethylated to form mono-, di-, and trimethyl Sb in the environment (Filella, 2010). Trimethyl Sb(V) (TMSb) was found to be the dominant Sb species in soil and in plant leaves in an Sb-mining area of China (Wei et al., 2015), and dimethyl Sb(V) (DMSb) was detected in liverworts and mosses growing close to an abandoned Sb mine in the UK (Craig et al., 1999). In soil, the origin of methylated Sb is attributed to microbial activity (Bentley and Chasteen, 2002). If Sb biomethylation includes changes in oxidation state, as in the case of As methylation according to Challenger's theory (Challenger, 1945), then a change in redox potential should affect biomethylation and thereby influence the solubility of Sb in soil. Frohne et al. (2011) suggested that low redox potential promotes the mobility of methylated Sb, based on the observation that the concentrations of mono- and di-methylated Sb decreased linearly with increasing redox potential in a soil suspension. Grob (2016) detected TMSb in the pore water of shooting range soils after 4 days of waterlogging, and Yang and He (2016) found higher concentration of methylated Sb in paddy soils than in dryland soils. Furthermore, the volatile compound trimethylstibine was found in sewage sludge sampled from anaerobic wastewater (Michalke et al., 2000). While these studies indicate that methylated species may play a relevant role in Sb turnover in the environment, the work by Mestrot et al. (2016) is the only published study so far on TMSb uptake and translocation in plants. As well as investigating TMSb they also detected traces of TMSb in roots and shoots of ryegrass plants treated with Sb(III) and Sb(V).

While there is a lack of knowledge about the interactions between methylated Sb and plants, investigations of the uptake and turnover of methylated As by plants may give some hints, based on the fact that Sb and As are sister elements in the periodic table and similar in many chemical characteristics. Zhao et al. (2013) found inorganic As, monomethylarsinic^V^ acid (MMA) and dimethylarsinic^V^ acid (DMA) to be the dominant As species in paddy-grown rice, while (Ma et al., 2016) found that the main species in *Panax notoginseng* collected from the field were As(III) and MMA, although the main species in the soils was arsenate (As(V)). Given that TMSb was found to be more mobile than methylated arsenate in soils (Yang and He, 2015), transfer of methylated species from soils into plants may also be a relevant process for Sb in soil under low-redox conditions. Moreover, it is not known whether Sb methylation may also occur in plants. Methylation may lead to profound changes not only in the mobility of Sb, but also in its toxicity, as is the case for As (Dopp et al., 2010).

Using a recently developed method of chemical extraction and high-performance liquid chromatography inductively coupled plasma mass spectrometry (HPLC-ICP-MS) analysis for the identification of Sb species (Mestrot et al., 2016), we addressed two questions in the present study: (1) How does accumulation of methylated Sb in plants and its transformations, relate to the speciation of Sb in the solution to which the roots are exposed? (2) Can methylation of Sb occur inside plants? To answer these questions, we investigated the accumulation, translocation and transformation of inorganic and organic Sb species in a variety of agricultural plant species under hydroponic conditions. Due to the fact that many Sb contaminated sites are used for agriculture, it is important to assess the risks of Sb transfer from such soils into food and feed crop plants, and therefore to know how Sb accumulation, allocation and speciation in crop plants relates to Sb speciation in the soil.

## Materials and methods

All chemicals were of analytical or superpure grade. Nanopure water (18 MΩ.cm) was used for preparing stock solutions. All plastic and glass containers were acid washed before use. The plant species used in the experiment were: ryegrass (*Lolium perenne* L. Calibra), fescue (*Festuca pratensis* Huds. Preval), wheat (*Triticum aestivum* L. Sella), and rye (*Secale cereale* L. Palazzo). The seeds were obtained from Fenaco Genossenschaft and Delley Samen und Pflanzen, Switzerland. Ryegrass and meadow fescue are common grasses in Swiss agricultural grasslands, including shooting ranges used as pasture during times of no shooting activity, while wheat and rye were included for comparison and because they are widely cultivated cereals for food production.

### Experimental set-up

The seeds of the experimental plants were rinsed with 10% H_2_O_2_ for 15 min, then washed with nanopure water and germinated on moist rolled tissue. After germination, all plants were transferred (three replicates per species) to opaque 1-L bottles (10 plants per bottle for ryegrass and fescue, 1 plant per bottle for wheat and rye) filled with continuously aerated 20% Hoagland nutrient solution (Hoagland and Arnon, 1950), buffered with 2-(N-morpholino)ethanesulfonic acid (MES) at pH 6 in a climate chamber. The climate chamber had a daily photo period of 16 h at 22°C with 230 μmol m^−2^ s^−1^ photon flux and a daily night period of 8 h at 16°C. The plants were grown for 4 weeks, and the nutrient solutions were changed twice per week during this time. After 4 weeks, the same nutrient solutions containing 1 mg L^−1^ Sb(III), Sb(V), or TMSb were supplied to the plants for 8 days. The Sb nutrient solutions were prepared from 1,000 mg L^−1^ Sb stock solutions prepared from either dissolved KSbOH_6_ for the Sb(V) treatment, Sb_2_O_3_ in 2 M HCl (Merck Millipore) for the Sb(III) treatment, or dissolved trimethyl Sb(V) bromide (Sigma-Aldrich) for the TMSb treatment. The same amount of HCl as present in the Sb(III) nutrient solution was added also to the Sb(V) and TMSb nutrient solutions. The pH was adjusted to pH 6 using 2 M KOH. The nutrient solutions containing Sb(III) were not aerated, whereas the Sb(V) and TMSb solutions were aerated during the experiment. During Sb exposure, the solutions were changed every 2 days and sampled before plant introduction and after 2 days of plant growth to analyze total Sb concentrations and Sb speciation (Mestrot et al., 2016). No TMSb was found in the inorganic Sb nutrient solutions and no inorganic Sb was found in the TMSb nutrients solutions. After harvest, all plant roots and shoots were washed, shock frozen in liquid nitrogen, freeze dried, and ground under anoxic conditions. The dry plant materials were kept in a N_2_-filled glovebox till extraction. The abbreviations used in figures and tables of this article are listed in Table 1.

**Table 1:** Abbreviations used to denote the 12 combinations of experimental plant species and Sb treatments in this study.

**Treatment**	**Sb(III)**	**Sb(V)**	**TMSb**
Wheat	WIII	WV	WTM
Fescue	FPIII	FPV	FPTM
Rye	RIII	RV	RTM
Ryegrass	GIII	GV	GTM

### Microwave digestion and element analysis

Aliquots of 100 mg ground plant material were digested in 1 mL 65% HNO_3_ and 2 mL 30% H_2_O_2_ in a closed microwave system (Turbowave 1500, MLS GmbH). Certified reference materials (Leyland cypress, IPE 171, WEPAL, Wageningen, recovery rate = 98.1 ± 2.3%, *n* = 3) and blanks were digested with each batch. After cooling to prevent loss of volatile Sb, samples were made to a volume of 10 mL with nanopure water and stored at 4°C until analysis.

For inductively coupled plasma mass spectrometry (ICP-MS, Agilent 7900, Agilent Technologies) measurement of Sb, the digests were diluted with a final 0.2% HCl concentration in solutions and internal standards were mixed with samples online during injection (10 μg L^−1^ holmium, yttrium, indium, lutetium). The nutrient solutions, which were stored in 1% HNO_3_, were diluted with nanopure water and analyzed by ICP-MS for total Sb.

Translocation factors (TFs) of total Sb in different treatments and different Sb species in different plant species were calculated as below

(1) $$\begin{array}{r} TF=\;\frac{Sb\;concentration\;in\;plant\;shoots\;\left(mg{\;kg}^{-1}\;DW\right)\;}{Sb\;concentration\;in\;plant\;roots\;\left(mg{\;kg}^{-1}\;DW\right)} \end{array}$$

### Sb extraction and Sb speciation

The Sb in plant shoots and roots was extracted based on the method developed by Mestrot et al. (2016). Briefly, 100 mg dry plant material was weighed into a 20 mL glass vial and mixed thoroughly with 10 mL 200 mM oxalic acid and 100 mM ascorbic acid solution in an ultrasonic bath. After 30 min, the vials were centrifuged at 3,500 rpm for 5 min. The supernatant was separated and filtered with 0.45 μm PTFE filter and stored at 4°C until analysis.

HPLC-ICP-MS (Agilent 7700x with Agilent 1200 HPLC) was used for measuring inorganic Sb(III), Sb(V), and TMSb coupled with a Hamilton PRP-X100 column. The extracts were diluted with nanopure water. The calibration standards were prepared daily by diluting the Sb stock solution with 150 mM ammonia tartrate (≥99.5%, Sigma-Aldrich). The mobile phase was 150 mM of ammonium tartrate at pH 5 with 4% methanol at a flow rate 1 mL min^−1^ (Mestrot et al., 2016). At sampling the nutrient solution samples were diluted with nanopure water and stored at 4°C until analysis. Just prior to speciation analysis they were diluted 10 times with 150 mM ammonium tartrate and analyzed by the same method for Sb speciation as plant extracts. The limit of detection (LODs) of Sb(III), Sb(V), and TMSb were 0.5 μg L^−1^.

### Statistics

Differences between treatments of a given plant species and between plant species in a given Sb treatment were tested by means of one-way ANOVA followed by multiple comparisons with Tukey's HSD-test using IBM SPSS Statistics 22. Differences between means were considered significant for *p* ≤ 0.05.

## Results

### Total Sb in plants and ascorbic acid/oxalic acid extracts

**Figure 1:**
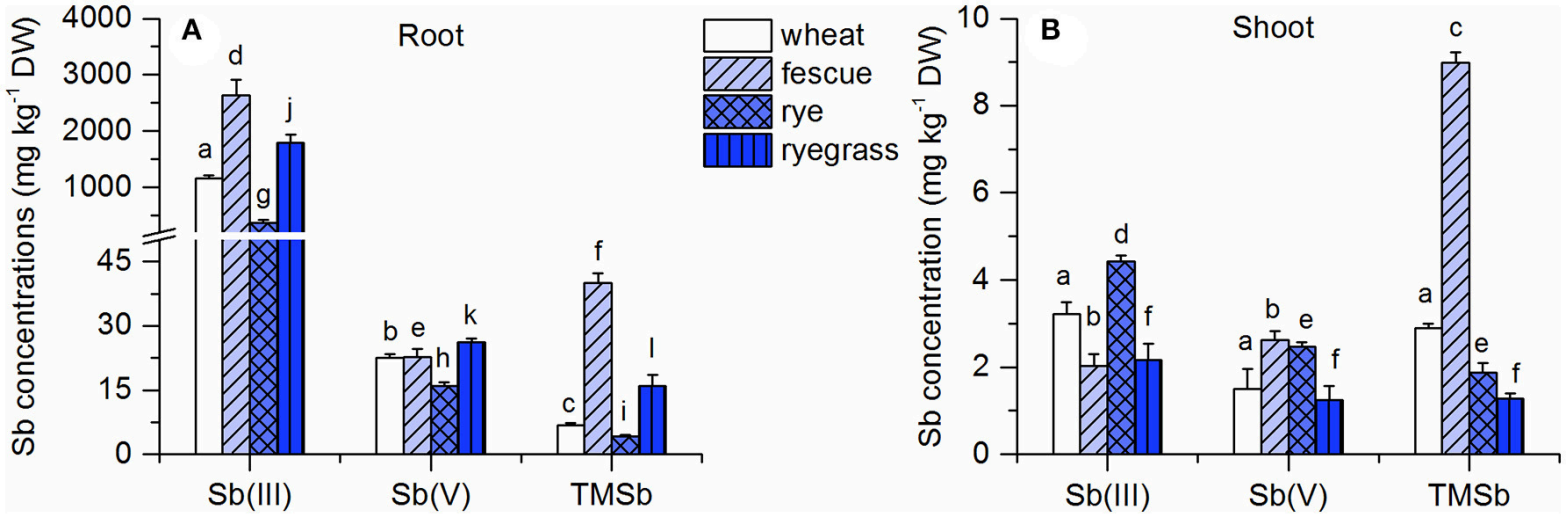
Total Sb concentrations in **(A)** roots and **(B)** shoots of the four plant species in the Sb(III), Sb(V), and TMSb treatments. The letters represent statistical comparisons of Sb treatments only within each plant species (mean ± SE, *n* = 3).

Plants tended to accumulate much more Sb in their roots (>350 mg kg^−1^) in the Sb(III) treatment than in the other Sb treatments (<40 mg kg^−1^) (Figure 1). Furthermore, the root Sb concentrations of wheat, rye, and ryegrass were significantly higher in the Sb(V) treatment than in the TMSb treatment. Only the roots of fescue contained less Sb in the Sb(V) treatment than in the TMSb treatment. Compared to the root Sb concentrations, shoot Sb concentrations were low (1–9 mg kg^−1^) and showed no significant variability between treatments, except in fescue, which took up significantly more Sb in the TMSb treatment than in the inorganic Sb treatments.

TFs for the three Sb treatments followed the order TMSb > Sb(V) > Sb(III) in all four plant species (Table S1). The TFs were at least 10 times larger in the Sb(V) treatment than in the Sb(III) treatment and around 2 times larger in the TMSb treatment than in the Sb(V) treatment.

**Figure 2:**
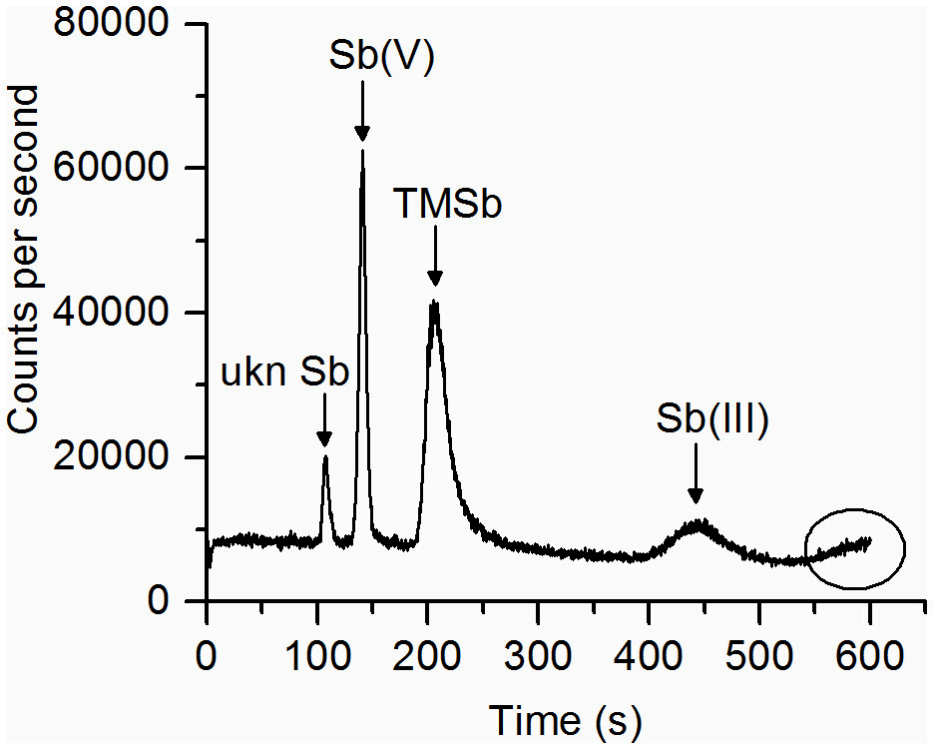
Chromatogram of a ryegrass root sample extract showing a peak of an unknown Sb species (ukn Sb) before the peaks of Sb(V), TMSb, and Sb(III) appeared. At the end of measurement, a broad peak tended to elute.

Our extraction method (ascorbic acid/oxalic acid solution) extracted 70–98% of the total Sb contained in the plant samples. The overall extraction efficiency did not differ between Sb treatments and plant species (Table S2). For some samples, the Sb species concentrations measured by HPLC-ICP-MS (peaks appearing in the chromatograph), added up to much less than the total Sb concentrations in the ascorbic acid/oxalic acid extracts (measured by ICP-MS). This indicates that not all Sb species in the extracts were separated by the HPLC-ICP-MS method (HPLC efficiency 30–95%, Table 2). These Sb species were regarded as non-eluted Sb. In particular, there was more non-eluted Sb in the roots of fescue and ryegrass in the TMSb treatment than that in the Sb(III) and Sb(V) treatments (Table 2). Furthermore, as shown in Figure 2, one Sb species showed up as a nicely separated chromatographic peak, but remained unknown due to a lack of standards. This unknown Sb species (ukn Sb) was found in all plants in the TMSb treatment, but not in the other treatments. For plant shoots, the HPLC efficiency was always around 95% and did not differ between Sb treatments.

**Table 2:** HPLC-ICP-MS speciation of Sb % of total Sb in the roots and shoots of four plant species in three Sb treatments [III, Sb(III) treatment; V, Sb(V) treatment; TM, TMSb treatment].

**Treatment**	**Root**					**Shoot**				
	**Sb(V)**	**Sb(III)**	**TMSb**	**ukn Sb**	**Non-eluted Sb**	**Sb(V)**	**Sb(III)**	**TMSb**	**ukn Sb**	**Non-eluted Sb**
Wheat III	6.3 ± 1.1	75.9 ± 0.9	< LOD			47.2 ± 4.6	36.1 ± 5.5	1.7 ± 0.1		
Fescue III	4.2 ± 0.4	61.7 ± 6.2	< LOD		4.8 ± 1.9	56.5 ± 1.1	17.0 ± 1.1	4.3 ± 0.7		2.9 ± 0.8
Rye III	5.3 ± 0.1	71.4 ± 1.2	< LOD		4.6 ± 2.2	33.6 ± 3.5	47.5 ± 2.9	1.5 ± 0.0		1.7 ± 0.4
Ryegrass III	5.3 ± 0.3	75.7 ± 0.9	< LOD		5.0 ± 0.9	43.5 ± 7.8	30.6 ± 5.4	3.9 ± 0.2		
Wheat V	90.7 ± 0.6	3.4 ± 0.6	0.8 ± 0.1			60.6 ± 13.6	< LOD	4.6 ± 1.6		3.84 ± 1.7
Fescue V	87.3 ± 1.8	1.3 ± 0.1	0.7 ± 0.0			78.5 ± 1.3	< LOD	5.4 ± 0.5		
Rye V	95.0 ± 1.5	3.9 ± 1.0	1.1 ± 0.0			83.5 ± 5.3	3.0 ± 0.1	3.1 ± 0.3		
Ryegrass V	87.8 ± 2.5	2.5 ± 0.8	0.9 ± 0.0			66.5 ± 14.8	< LOD	5.6 ± 1.0		
Wheat TM	13.6 ± 1.2	21.6 ± 5.3	35.9 ± 1.3	0.6 ± 0.0		1.9 ± 0.4	2.6 ± 0.1	86.8 ± 0.6	1.4 ± 0.0	
Fescue TM	4.7 ± 0.4	2.6 ± 0.9	26.0 ± 1.2	0.7 ± 0.1	41.1 ± 0.6	0.7 ± 0.0	< LOD	75.3 ± 2.8	2.6 ± 0.3	
Rye TM	7.8 ± 2.0	4.0 ± 0.2	47.3 ± 7.8	1.2 ± 0.2	18.5 ± 4.1	1.1 ± 0.1	< LOD	70.4 ± 13.9	2.2 ± 0.3	3.0 ± 1.3
Ryegrass TM	6.2 ± 0.7	2.6 ± 0.5	11.3 ± 0.8	0.8 ± 0.1	70.2 ± 1.4	8.2 ± 4.4	< LOD	61.9 ± 4.2	8.4 ± 0.5	3.5 ± 1.1

*Notation for Sb species: Sb(V), inorganic Sb(V); Sb(III), inorganic Sb(III); TMSb, trimethyl Sb(V); ukn, unknown Sb species. mean ± SE, n = 3, LOD, limit of detection*.

### Speciation of Sb in Sb(III) and Sb(V) treated plants

**Figure 3:**
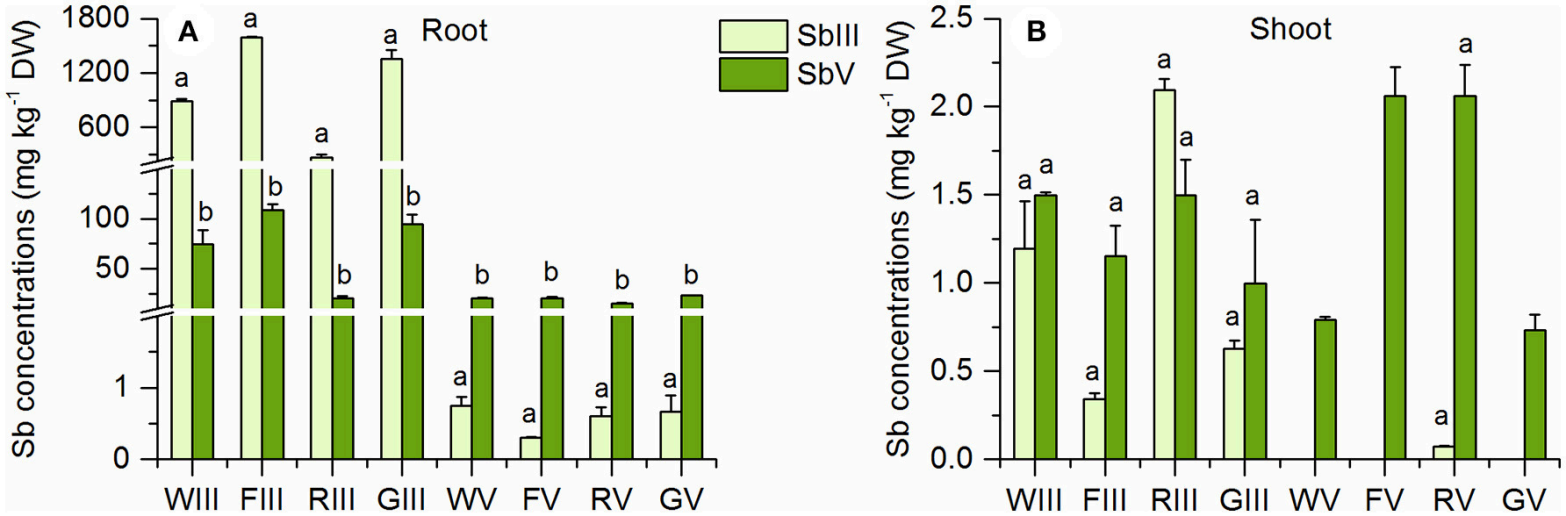
Sb(III) and Sb(V) concentrations in **(A)** roots and **(B)** shoots of the four plant species in the Sb(III) and Sb(V) treatments (mean ± SE, *n* = 3). The letters represent the statistical comparison of Sb species in each plant species/treatment combination.

The plants had different proportions of inorganic Sb species in their shoots and roots in the Sb(III) and Sb(V) treatments (Figure 3). In the Sb(III) treatment, plant roots contained 4–7% Sb(V) (Table 2), which was similar to the 5% Sb(V) in the samples of the initial nutrient solutions (Table S3). Because nutrient solution samples were diluted with nanopure water and stored at 4°C until analysis, some Sb(III) may have oxidized to Sb(V) in the Sb(III) nutrient solutions samples during storage. There was over 10 times more Sb(III) than Sb(V) in Sb(III) treated plant roots (Figure 3A). On the other hand, the concentrations of Sb(III) were not significantly larger than those of Sb(V) in the shoots of Sb(III) treated plants (Figure 3B). For wheat, fescue, and ryegrass, there was even slightly more Sb(V) in the shoots than Sb(III). Percentages of Sb(V) increased from around 5% in roots to over 30% in shoots (Table 2). In the Sb(V) treatment the dominant species in plant shoots and roots was Sb(V). In plant roots the percentage of Sb(III) was <4% while there was no detectable Sb(III) in plant shoots except for rye with 0.07 mg kg^−1^ (Table 2, Figure 3B).

**Figure 4:**
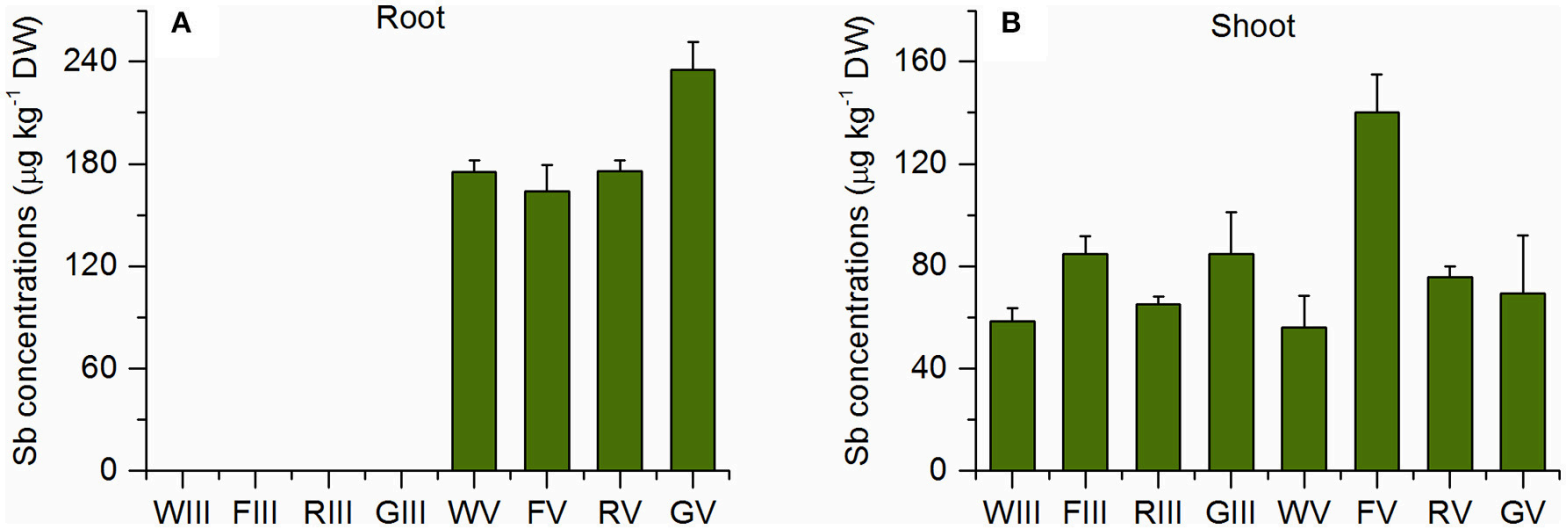
TMSb concentrations in **(A)** roots and **(B)** shoots of the four plant species in the Sb(III) and Sb(V) treatments (mean ± SE, *n* = 3).

Figure 4 shows that there were similar concentrations of TMSb in the roots of all Sb(V) treated plants. Also in the Sb(III) treatment, there appeared to be a small peak of TMSb (Figure S1). However, as the samples were diluted by a factor of 100 due to the high total Sb concentration, the peak area was below the LOD, 5 mg kg^−1^. The concentrations of shoot TMSb were very similar in all plant species with no difference between the two inorganic Sb treatments, except for Sb(V) treated fescue (Figure 4B). The TMSb percentages of total shoot Sb were slightly larger in the Sb(V) treatment than in the Sb(III) treatment for each plant species (Table 2).

### Speciation of Sb in TMSb treated plants

**Figure 5:**
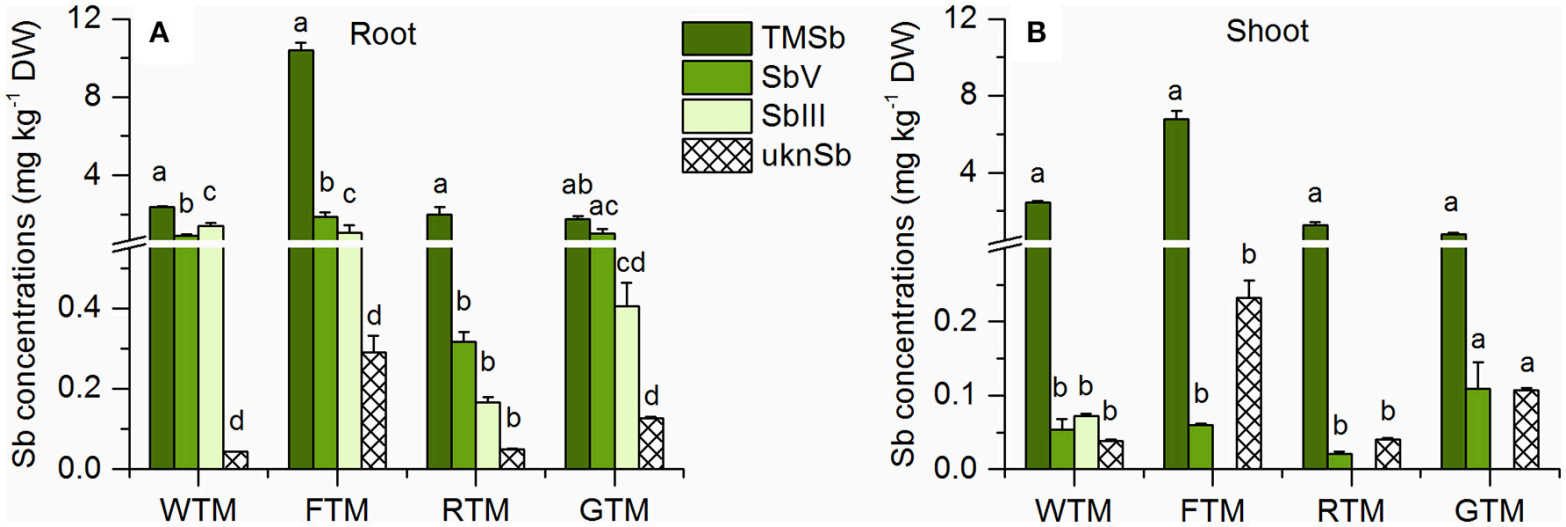
Sb species in **(A)** roots and **(B)** shoots for different plant species of the TMSb treatment (mean ± SE, *n* = 3). ukn Sb = peak of unknown Sb species in chromatograms of ascorbic acid/oxalic acid extracts. The letters represent the statistical comparison of Sb species concentrations for each plant species separately.

In addition to the three Sb species TMSb, Sb(V), and Sb(III), we also found the unknown species (ukn Sb) (as mentioned before) in the TMSb treated plants (Figure 5). The abundance of these four species decreased in the order TMSb > Sb(V) > Sb(III) > ukn Sb in the roots and shoots of all plants (Table 2). Comparing their concentrations among plant species, root, and shoot concentrations of TMSb and ukn Sb were highest in fescue, while they did not vary much among the other three plant species.

The TF of the ukn Sb species was >0.8 in all four plant species, which was higher than that of TMSb, except for wheat (Table 3). Both inorganic Sb species, Sb(III) and Sb(V) existed in the roots of the four plant species, but there were only small concentrations of Sb(V) and no Sb(III) in the shoots, apart from 0.07 mg kg^−1^ Sb(III) in wheat (Figure 5). Root Sb(III) concentrations were lower than root Sb(V) concentrations, except for wheat which had lower Sb(V) concentrations.

**Table 3:** Translocation factors (TFs) of TMSb, Sb(V), Sb(III), and ukn Sb in four plant species in the TMSb treatment.

**Sb species**	**Wheat**	**Fescue**	**Rye**	**Ryegrass**
TMSb	1.02	0.65	0.63	0.45
Sb(V)	0.059	0.031	0.064	0.107
Sb(III)	0.050	0	0	0
ukn Sb	0.90	0.80	0.82	0.84

## Discussion

### Sb accumulation in plant roots and translocation to plant shoots

Generally, Sb concentrations in the roots of wheat, rye, and ryegrass exposed to different Sb species followed the order TMSb treatment < Sb(V) treatment < Sb(III) treatment. The root total Sb concentrations measured in this experiment included both extracellular (apoplastic) and intracellular (symplastic) sources, as we digested the whole root without trying to remove apoplastic Sb. Thus, the different affinities of ions for root cell walls need to be taken into account to explain the treatment differences in root Sb concentrations in addition to differences in uptake pathways. The chemical speciation of Sb in aqueous solutions depends on pH and this may include the change of molecular charge of the redox species with pH. In this experiment, the pH of the nutrient solution was 6, and under such weakly acid condition the dominant Sb(III) species is the neutral solute Sb(OH)_3_, while Sb(V) exists as the monovalent oxyanion ${\text{Sb}(\text{OH})}_{6}^{-}$ (Vink, 1996; Ritchie et al., 2013). This explains higher root concentrations in the Sb(III) than in the Sb(V) treatment as resulting from the repulsion of ${\text{Sb}(\text{OH})}_{6}^{-}$ from charged cell walls (Marschner and Marschner, 2012). The Sb species present in the TMSb treatment were difficult to determine, as there is very little research on the solution chemistry of TMSb. It may be present in the form of the neutral species (CH_3_)_3_Sb(OH)_2_ or as the monovalent cation [(CH_3_)_3_SbOH]^+^ in aqueous solutions under neutral conditions (Zheng et al., 2001). Theoretically at least, the neutral (CH_3_)_3_Sb(OH)_2_ and the positively charged [(CH_3_)_3_SbOH]^+^ should be adsorbed more strongly on cell walls than ${\text{Sb}(\text{OH})}_{6}^{-}$, but nonetheless higher Sb concentrations were found in the Sb(V) treatment than in the TMSb treatment in the roots of wheat, rye, and ryegrass. This suggests that TMSb and Sb(V) use different uptake pathways.

Despite the differences in root Sb concentrations, the shoot Sb concentrations were quite similar in the three Sb treatments, which suggests that different Sb species have different TFs (Figure 1). As can been seen in Table S1, the TF for total Sb was always the highest in the TMSb treatment when the three Sb treatments are compared for the four different plant species. This suggests that TMSb was more mobile in the plants than inorganic Sb after uptake, which may have been related to different translocation pathways associated with the very different chemical nature of this organic species as compared to the inorganic species.

### Speciation of root and shoot Sb in the Sb(III) and Sb(V) treatments

In a similar manner to As(III), Sb(III) can be taken up into plant cells through transporters of the aquaporin family (Bienert et al., 2008; Kamiya and Fujiwara, 2009). In the Sb(III) treatment, more than 70% Sb found in the roots was Sb(III) and <6% was Sb(V). It is unlikely that substantial oxidation of Sb(III) occurred in the plant samples after harvesting, as the samples were processed under oxygen free conditions, and ascorbic acid and oxalic acid were used as reducing agents to prevent Sb oxidation in the extracts. While the freshly prepared Sb(III) nutrient solutions contained <5% Sb(V) and were renewed every 2 days, the proportion of Sb(V) increased up to 13% in the nutrient solution samples collected after 2 days immersion of plant roots. The increase of Sb(V) concentrations in the nutrient solutions could be due to water extraction by the plants for transpiration or oxidation of Sb(III) during plant growth or nutrient solution sample storage. Although the plants thus may have taken up some Sb(V) directly from the solutions in the Sb(III) treatments, this does not fully rule out Sb(III) oxidation inside the plants, however. Some studies provided evidence for oxidation of As(III) within plant tissues (Lombi et al., 2002; Tu et al., 2003; Wan et al., 2017), and due to the chemical similarity of Sb(III) and As(III), oxidation thus may occur in a similar manner also to Sb(III). Tu et al. (2003) grew *Pteris vittata* in soils spiked with As(V) and found that more than 95% of As in the young and mature fronds was As(III), but <80% in the old fronds. They suggested that the lower fraction of arsenite in the old fronds was due to re-oxidation as a result of reduced concentrations of ascorbate in the old fronds. The same conclusion was drawn by Lombi et al. (2002), who found that 35% of the As accumulated in the fronds of *P. vittata* treated with As(V) was present in the form of As(V), based on X-ray near-edge structure (XANES) spectrosocpy. Analogous results were obtained by Wan et al. (2017) recently. They found that exposure to As(III) for 7 days resulted in up to 40% of As(V) in the rhizoids of *P. vittata* plants. Furthermore, small concentrations of As(V) were found in rice plants treated with As(III) (Lomax et al., 2012)

As shown in Table 2, the percentages of Sb(V) increased from roots to shoots in the Sb(III) treatment, while those of Sb(III) decreased, suggesting that Sb(III) was retained in the roots more strongly than Sb(V). We see two main reasons that could account for this. The first could be that neutral Sb(OH)_3_ was bound to the root apoplast more strongly than the negatively charged ${\text{Sb}(\text{OH})}_{6}^{-}$, so that a smaller fraction was transferrable to the shoots. The second reason could be detoxification of Sb(III) through binding to thiol groups and transfer into root cell vacuoles. The mechanisms of Sb detoxification in plant cells are still not well understood. A phytochelatin synthase (AtPCS1) was found to convey As and Sb tolerance to *A. thaliana* plants (Kamiya and Fujiwara, 2011). Given that the chemistry of As is in many ways similar to that of Sb, the mechanisms involved in As(III) detoxification, complexation by phytochelatins, and transfer of the complexes into root cell vacuoles, may inhibit mobility and root-to-shoot translocation also in the case of Sb(III). In a recent synchrotron based study, more than 50% of Sb species in roots and shoots of ryegrass treated with Sb(III) were found to be Sb-thiol complexes (Ji et al., 2017). Although according to our speciation results, all Sb found in oxidation state III was in the inorganic form, it could be that Sb-thiol complexes in plant cells were either not extracted or that they dissociated when vacuoles broke during extraction (Bluemlein et al., 2009; Lombi et al., 2009).

The small amounts (<4%) of Sb(III) found in plant roots and shoots in the Sb(V) treatment could be due to chemical reduction during extraction. In a previous study, Mestrot et al. (2016) found that up to 28% of spiked Sb(V) was reduced to Sb(III) in the ascorbic acid/oxalic acid extraction solution. It is also possible that reduction of Sb(V) to Sb(III) happened in the plant, as there is also evidence for As(V) reduction in plants (Zhao et al., 2009; Kashiwabara et al., 2010). Because of the low concentrations of Sb(III) in our Sb(V) treated plants, the place where the reduction occurred is difficult to localize. More than 60% Sb was retained in the form of Sb(V) after uptake into the plants in the Sb(V) treatment (Table 2). As 13–30% Sb in the shoots was not extracted by the ascorbic acid/oxalic acid extraction method (Table S2), it is possible that some of Sb(V) was transformed to other compounds after translocation.

In addition to the dominant inorganic Sb species, also some TMSb (<6%) was found in the roots and shoots of Sb(III) and Sb(V) treated plants. TMSb has been detected also in plants and soils collected from an Sb-mining area in China (Wei et al., 2015). Again, there is an analogy with findings of methylated As in plants. Small amounts of methlylated As were found in plants collected from various field sites (Hansen et al., 2011; Ma et al., 2016). Sunflower (*Helianthus annuus*) plants grown in As(III) solution showed small concentrations of both MA and DMA, although they were difficult to quantify (Raab et al., 2005). DMA also was a major methylated As species in *H. annuus* grown in As(V) solution, although it amounted to only 1% of the total As concentration (Raab et al., 2007a). In contrast, rice, tomato and clover plants grown for more than 30 days in sterilized media containing inorganic As showed no trace of methylated As in their tissues (Lomax et al., 2012). Some authors performing similar studies to ours, but with As instead of Sb, argued that the methylated As found in their experimental plants originated from microbial methylation in the growth media (Zangi and Filella, 2012; Jia et al., 2013; Zhao et al., 2013). Although the solutions were not sterilized in our experiments, there was no detectable TMSb in the nutrient solutions used in the Sb(III) and Sb(V) treatments. While we cannot exclude that some TMSb was taken up from TMSb present in the solutions at concentrations below the detection limit, we also cannot rule out that some Sb was methylated inside the plants. The latter would not necessarily mean that it happened in plant cells. It is well known that plants are colonized by a large variety of microorganisms, collectively known as endophytes, which can perform important functions in plants. It is thus possible that also Sb methylation in plants could be actually due to microbial activity. Antimony biomethylation is commonly observed in microorganisms, i.e., bacteria and fungus (Bentley and Chasteen, 2002; Thayer, 2002). Wehmeier and Feldmann (2005) found that 0.8% of Sb added as isotopically labeled Sb(V) was converted to mono-, di-, and trimethyl Sb forms following the Challenger pathway in incubated sewage sludge obtained from anaerobic wastewater treatment. Antimony methylation in plants may follow a similar pathway of successive methylation steps. But with only one published study so far about Sb methylation in plants (Mestrot et al., 2016), more work is required to identify the mechanisms resulting in the presence of TMSb in plants in the absence of root exposure to detectable methylated Sb concentrations in the growth medium.

### Speciation of Sb in the TMSb treated plants

The results obtained from the TMSb treatment indicate that plants can take up substantial amounts of TMSb, but they do not give information about the uptake pathway. To the best of our knowledge, there has been no research on this subject. Some clues may come again from looking into research on methylated As in plants for enlightenment. Li et al. (2009) discovered that the silicon transporter Lsi1 (aquaporin, NIP 1;2), which is known to be involved in arsenite uptake, can also mediate the uptake of undissociated methylated As in rice. It has also been found that MMA and DMA were taken up into rice roots via the same pathway as glycerol, i.e., through aquaporins, as increased glycerol concentrations significantly inhibited their uptake (Rahman et al., 2011). Even though little is known about the solution chemistry of TMSb, it thus seems possible that TMSb was taken up via an aquaporin family based transporter in a similar manner to As(III) and Sb(III).

The shoot-to-root concentration ratio was at least four times higher for TMSb than for inorganic Sb in the TMSb treatment in all four experimental plants (Table 3), suggesting that TMSb is more mobile in plants than inorganic Sb species. The finding is in line with similar findings relating to As in plants. Investigating the concentrations of arsenate, MMA and DMA in the roots and shoots of 46 plant species, Raab et al. (2007b) found that the median TF of DMA was nearly three times larger than the TF of MMA and 10 times larger than the TF of arsenate. Likewise, Jia et al. (2012) found in a field experiment with rice that the root-to-shoot TFs for methylated As were higher than for inorganic As and that they increased with increasing degree of methylation.

The finding of inorganic Sb species in the TMSb treated plants, although the nutrient solutions contained no detectable trace of other Sb species than TMSb in this treatment, indicates that some TMSb was transformed to inorganic Sb after uptake by the plants. Analogous results were obtained by Mishra et al. (2017) with As. They found inorganic As species apart from DMA in the shoots of DMA-treated rice plants, while the roots in addition contained detectable amounts of MMA and trivalent methylarsonous acid (MA^III^). There is evidence that DMA can be demethylated to MMA in rice roots (Lomax et al., 2012). The amounts of As species with lesser degrees of methylation than DMA in DMA treated plants was found to show large variability among different plant species. Huang et al. (2008) applied XANES spectroscopy to three hyperaccumulating and hypertolerant plants treated with DMA. While DMA was found to be the major compound in the rhizoids, petioles and pinnae of *P. vittata* as well as in stems and leaves of *Boehmeria nivea*, but the dominant As species in the roots of *B. nivea* was As(III)-GSH, and in *P. cretica* As(III) was the dominant As species in all parts of the plants. While the mechanisms underlying the transformations of As in plants are not known, the fact that different plant species showed different proportions of the various inorganic and organic As species in their tissues suggests that plants differ widely in their capacity for transformation of methylated As and this may also be the case for Sb.

Yan et al. (2015), who investigated the demethylation of MMA in the cyanobacterium *Nostoc*, concluded that the bacteria first reduce MMA to MMAs(III) and then demethylate MMAs(III) to As(III). Assuming that the transformations of methylated Sb and As follow analogous pathways, the most obvious hypothesis explaining our results would be that TMSb was demethylated in a step-by-step demethylation process from TMSb through dimethyl and monomethyl Sb to inorganic Sb(V), which as then reduced to Sb(III). This process may help detoxify plant Sb, as Sb(III) can be bound to other functional groups. A similar detoxification mechanism has been described for As by Huang et al. (2008). However, these are just suggestions, and it is also possible that TMSb demethylation follows other reaction pathways.

While the low percentages (<4%) of Sb(III) in the roots of fescue, rye, and ryegrass could have been due to reduction by the extraction solution, the high percentage (21.6%) of Sb(III) in the wheat roots provides evidence that reduction of Sb(V) to Sb(III) also occurred in the TMSb treatment before sample preparation, at least in one plant species. The fact that there was no trace of Sb(III) in the shoots of fescue, rye, and ryegrass but a small amount of Sb(III) in shoots of wheat lends further supports to this assertion. The only previous study on Sb speciation in plants treated with methylated Sb also found some Sb(V) in ryegrass roots and shoots, but no Sb(III) (Mestrot et al., 2016).

The unknown Sb species detected in the TMSb treated plants could not have been inorganic Sb. Given that its peak was close to TMSb in the chromatogram, we hypothesize that it was another organic Sb species. Based on its shoot-to-root concentration ratios, it seemed to be even more mobile than TMSb.

With its low LOD for TMSb the analysis method applied in this study is ideal for measuring low TMSb concentrations in the roots and shoots of plants exposed to inorganic Sb. However, there is still a risk of Sb species conversion during chemical extraction. Small amounts of TMSb were found in the roots and shoots of the plants in both inorganic Sb treatments, suggesting that methylation might have occurred inside the plants or in the zone known as rhizosphere around plant roots, where microbial activity is greatly enhanced by root exudates. This means that there may be a risk that methylated Sb could accumulate in plants which are growing on contaminated soils, even if the dominant Sb species are inorganic in the soil. Although uptake of TMSb was lower than uptake of inorganic Sb, TMSb was more readily translocated to plant shoots. This might cause health risks for animals or humans consuming the aboveground parts of crop plants grown on such soils, especially considering that methylated Sb might be more toxic than inorganic Sb, as was found with arsenic (Bentley and Chasteen, 2002). Potential transformation of TMSb into other Sb species including organic compounds that can be more mobile in plant tissues than TMSb, just adds another component of uncertainty to such risk assessments. The results of our study thus call for further investigation of the soil-to-plant transfer of different Sb species and their transformation reactions and mobility within plants.

## Author contributions

ST and RS supervised the Ph.D. project of YJ. YJ designed the experiment with the help of ST and RS and carried out the experiment. AM developed the analysis method applied in the experiment and helped the analysis of extracts from plant samples. YJ wrote the manuscript with the support from ST, RS, and AM. All authors discussed the results and contributed to the final manuscript.

### Conflict of interest statement

The authors declare that the research was conducted in the absence of any commercial or financial relationships that could be construed as a potential conflict of interest.
