# Supplementary material for: Uptake and Transformation of Methylated and Inorganic Antimony in Plants
Source: Front Plant Sci. 2018 Feb 13;9:140. doi: 10.3389/fpls.2018.00140 (PMC5816898; doi:10.3389/fpls.2018.00140)
Supplement: Supplementary file 1 [file Table1.DOCX]

TABLE S1. Translocation factors of total Sb in four plant species in three Sb treatments.

| Treatment | Wheat |  | Fescue |  | Rye |  | Ryegrass |
| --- | --- | --- | --- | --- | --- | --- | --- |
| Sb(III) | 0.0028 |  | 0.0008 |  | 0.012 |  | 0.0012 |
| Sb(V) | 0.067 |  | 0.115 |  | 0.154 |  | 0.047 |
| TMSb | 0.423 |  | 0.224 |  | 0.450 |  | 0.080 |
