# Supplementary material for: Uptake and Transformation of Methylated and Inorganic Antimony in Plants
Source: Front Plant Sci. 2018 Feb 13;9:140. doi: 10.3389/fpls.2018.00140 (PMC5816898; doi:10.3389/fpls.2018.00140)
Supplement: Supplementary file 2 [file Table2.DOCX]

TABLE S2. Percentages of extracted Sb from plant roots and shoots in three Sb treatments. Mean ± SE, n = 3.

| Treatment | Extraction % | |
| --- | --- | --- |
|  | Root | Shoot |
| Wheat III | 80.4 ± 3.4 | 81.0 ± 1.0 |
| Fescue III | 70.7 ± 5.1 | 80.7 ± 1.4 |
| Rye III | 81.3 ± 2.4 | 84.3 ± 1.7 |
| Ryegrass III | 86.1 ± 1.2 | 78.2 ± 3.7 |
| Wheat V | 90.8 ± 1.9 | 69.1 ± 16.7 |
| Fescue V | 86.6 ± 0.9 | 84.6 ± 2.2 |
| Rye V | 97.9 ± 2.0 | 86.8 ± 5.4 |
| Ryegrass V | 86.2 ± 2.5 | 72.7 ± 14.6 |
| Wheat TM | 73.8 ± 4.6 | 86.3 ± 1.3 |
| Fescue TM | 75.2 ± 2.1 | 75.5 ± 2.7 |
| Rye TM | 78.8 ± 1.2 | 76.8 ± 13.2 |
| Ryegrass TM | 91.1 ± 2.1 | 82.0 ± 3.9 |
