# Supplementary material for: Uptake and Transformation of Methylated and Inorganic Antimony in Plants
Source: Front Plant Sci. 2018 Feb 13;9:140. doi: 10.3389/fpls.2018.00140 (PMC5816898; doi:10.3389/fpls.2018.00140)
Supplement: Supplementary file 3 [file Table3.DOCX]

TABLE S3. Sb(V) percentage in nutrient solutions of the Sb(III) treatment before (initial solutions) and after plant introduction (wheat, fescue, rye and ryegrass).

|  | 0 day | 2nd day | 6th day | 8th day |
| --- | --- | --- | --- | --- |
| Initial solutions | 5.4% |  | 5.2% |  |
| Wheat |  | 12.4% |  | 8.9% |
| Fescue |  | 6.7% |  | 6.9% |
| Rye |  | 13.5% |  |  |
| Ryegrass |  | 7.1% |  |  |
